# Dogs and the Good Life: A Cross-Sectional Study of the Association Between the Dog–Owner Relationship and Owner Mental Wellbeing

**DOI:** 10.3389/fpsyg.2022.903647

**Published:** 2022-07-18

**Authors:** Aikaterini Merkouri, Taryn M. Graham, Marguerite Elizabeth O’Haire, Rebecca Purewal, Carri Westgarth

**Affiliations:** ^1^School of Veterinary Science, Institute of Infection, Veterinary and Ecological Sciences, University of Liverpool, Neston, United Kingdom; ^2^Department of Livestock and One Health, Institute of Infection, Veterinary and Ecological Sciences, University of Liverpool, Neston, United Kingdom; ^3^Department of Comparative Pathobiology, College of Veterinary Medicine, Purdue University, West Lafayette, IN, United States

**Keywords:** dogs, ownership, pets, human–animal bond, depression, mental health, anxiety, qualitative

## Abstract

Dog ownership is believed to benefit owner wellbeing but, contrary to popular belief, there is limited evidence to suggest that simply owning a dog is associated with improved mental health. This mixed-methods study investigates whether dog owners with stronger relationships with their dogs experience better mental health. Participants (*n* = 1,693, adult United Kingdom dog owners) completed an online survey. Owners’ health was measured using the validated PROMIS questions regarding depression, anxiety, emotional support, and companionship. The dog–owner relationship was measured using the validated MDORS scale, which has three subscales: interaction, emotional closeness, and perceived costs. Univariable and multivariable linear regression analyses were conducted, adjusting for confounding factors. Additionally, positive and negative impacts of dog ownership on mental wellbeing were coded from open questions using thematic analysis. A stronger dog–owner relationship was associated with greater feelings of emotional support and companionship but poorer mental health in terms of anxiety or depression. However, the perceived costs (burden) subscale was consistently associated with better mental health outcomes. Direction of causality cannot be inferred as people with poor mental health may acquire dogs to help relieve symptoms, which qualitative analysis supported. Key themes included positive impacts on owner wellbeing and happiness through providing purpose, companionship and self-acceptance, pleasure and distraction, as well as lessening emotional pain and suffering and reducing risk behaviors. However, negative impacts of a strong relationship include anticipatory grief over loss of the dog, and concerns regarding the burden of responsibility and ability to meet dog’s needs. Perceived ability to adequately meet dog’s needs promoted personal growth and positive relationships with others, whereas perceived inability led to feelings of guilt, or anger/frustration, and reduced autonomy and sense of environmental mastery. Dog ownership contributes to both hedonic and eudaimonic wellbeing in multiple ways, including supporting owners through periods of poor mental health and providing purpose. However, the burden of responsibility and owner and dog characteristics can create challenges, and owners may benefit from support in caring for their dogs and reducing problematic behaviors.

## Introduction

Mental health is “a state of wellbeing in which the individual realizes his or her own abilities, can cope with the normal stresses of life, can work productively and fruitfully, and is able to make a contribution to his or her community, “according to the World Health Organization (WHO, 2018b). At any time, 1 in 10 people worldwide are affected by a mental health disorder, bringing the absolute number to 792 million (Ritchie and Roser, 2018). Mental health conditions include depression, anxiety, obsessive–compulsive disorder and schizophrenia. Globally, in 2016, depressive symptoms are in the 16th place for Disability-Adjusted Life Year(s) (DALYs), and are in the top 20 DALYs for all regions and continents other than Africa (WHO, 2018a). Self-harm is included in the top 20 DALYs for all regions other than Africa and Eastern Mediterranean. Moreover, anxiety disorders are included in the top 20 DALYs list for the American continent (WHO, 2018a). In England, 1 in 4 adults encounters a mental health condition in any given year (Nice, 2019). From a financial point of view, the consequences of poor mental health were responsible for the decrease of the United Kingdom GDP by £25 billion in 2015 (Oxford Economics, 2016).

Many studies have suggested a positive association between interacting with an animal and psychological wellbeing (Barker and Wolen, 2008; Gilbey and Tani, 2015; Rodriguez et al., 2021). Studies of the impact of actual pet ownership, in contrast to interacting with a trained therapy or assistance animal, are less clear. Contrary to popular belief, most studies conducted comparing dog owners and non-dog owners found no association between dog ownership and improved mental wellbeing, as a result of inconsistent methodology, complexity of the human–animal interaction (HAI) relationship (Rodriguez et al., 2021), and demographical heterogeneity of pet owners (Fraser et al., 2020). In some cases, pet owners have reported more depressive symptoms than non-pet owners (Parslow et al., 2005; Enmarker et al., 2015; Fraser et al., 2020; Sharpley et al., 2020), and in particular owners with less friendly and less obedient pets experience higher depressive and anxiety symptoms, respectively, (Bradley and Bennett, 2015). Similarly, no evidence of change in depressive symptoms or positive/negative affect has been seen in longitudinal analyses of pet acquisition (Powell et al., 2019; Sharpley et al., 2020). Contrarily, in a study of treatment-resistant depression, it was found that an intervention group who adopted a pet improved compared to a control (Mota Pereira and Fonte, 2018), and in a study conducted on US veterans suffering from PTSD, it was reported that adopting a companion dog increased their wellbeing while decreasing their mental health symptoms (Stern et al., 2013). Further, among people with PTSD, owning a pet at the time of the traumatic event was associated with higher levels of happiness (González-Ramírez et al., 2019). There is also some evidence that dog owners may be less lonely (Gilbey and Tani, 2015; Powell et al., 2019) and less likely to report a long-standing mental health illness (Liu et al., 2019).

Studies of how the quality of the pet-owner relationship may moderate impacts on the owner’s wellbeing are even less clear. Some research suggests that a stronger attachment to a pet is associated with poorer mental health of the owner (Peacock et al., 2012). On the other hand, in pet owners with long-term mental health conditions, pets are considered to support their owner through encouraging social contact (Zimolag and Krupa, 2009), and distraction from the owner’s problems, thus pets are considered a main source of support (Brooks et al., 2016). Consequently, even though pets do not cure mental health conditions, they may help prevent or reduce symptoms (Hawkins et al., 2021). Most activities with a dog are perceived to have a positive effect on the owner psychological wellbeing, e.g., meeting with other people while outside with the dog increases owner’s positive relations with others, even if negative impacts also exist, for example, dog’s aging/death, dog’s unwanted behaviors or a perceived failure to meet dog’s needs (Barcelos et al., 2020).

Overall, findings are contradictory and complex, which may be because pet owners and non-pet owners also differ in many socio-demographic variables, which may influence the psychological profile of the individual, regardless of pet ownership (Saunders et al., 2017; Wong et al., 2019). The impact of dog ownership may also be influenced by the activities performed with the dog, for instance variable participation in dog walking (Westgarth et al., 2017), which is itself influenced by socio-demographic factors (Westgarth et al., 2014, 2017). In addition, direction of causality is difficult to infer from cross-sectional studies—for example, do dogs make people more depressed, or are depressed people more likely to seek comfort in dog ownership?

Furthermore, the study of human mental wellbeing is itself difficult to measure. What does it mean to “live well” or to have a “good life”? A key interest in psychology is answering these very questions through the lens of happiness, which can be split into two broad concepts: hedonism and eudaimonism (Ryan and Deci, 2001). Hedonism is based on the presence of positive affect and the absence of negative affect. Eudaimonism is focused on living life with meaning and purpose and can be categorized further into 6 elements: (1) autonomy (self-determining and independent), (2) personal growth (feeling of continued development), (3) self-acceptance (positive attitude toward self), (4) life purpose (goals and sense of direction), (5) environmental mastery (competence in managing the environment), and (6) positive relations with others (satisfying and trusting relationships; Ryff, 1989). The theoretical construct of hedonic and eudaimonic happiness has been used in qualitative research to explore how activities of dog ownership may impact on owner wellbeing, claiming both types of happiness are at play (Barcelos et al., 2020). However, the deductive approach used, by asking owners to view their interactions with their dogs through this explanatory framework and to suggest which activities fit into which concept, may not be an ideal method in comparison to an inductive approach where themes and theory are developed from the owner’s suggestions without supplying them with preconceived ideas (Pope et al., 2000).

In summary, the association between dog ownership and owners’ mental wellbeing requires further investigation. Little emphasis has been placed on the strength of the relationship between dogs and their owners and the impact this may have on potential wellbeing effects; the strength of the pet-owner relationship is not just about the amount of time spent together, but what is done during that time and the feelings that are developed toward the animal. Therefore, the first aim of this study was to investigate the association between the strength and dimensions of the dog–owner relationship and the owner’s psychological health outcomes. The second aim of the study was to inductively explore how aspects of the dog–owner relationship impact positively and negatively on the owner’s mental wellbeing, to complement the quantitative approach and so that the meanings and experiences behind directions of causality can be better understood (Pope and Mays, 1995).

## Materials and Methods

### Data Collection

An anonymous convenience sampling survey was conducted using Qualtrics online survey software from December 21, 2017 to February 9, 2018. Participants (United Kingdom resident dog owners aged 18 years or over) were recruited *via* social media advertising on Facebook and Twitter. The study was ethically approved by the University of Liverpool Veterinary Research Ethics Committee (Study VREC605) and participants provided informed consent by completing the survey after reading an information sheet.

### Questionnaire

The questionnaire consisted of five blocks of questions which included both closed and open-ended questions (see Supplementary Materials—Questionnaire). The first block comprised the eligibility questions (i.e., United Kingdom citizens, aged 18 or over, who consider themselves dog owners). The second block went by the name of “Dog-related questions,” including the dog’s demographics (see Table 1) and reasons for acquiring the dog. Participants who owned more than one dog were asked to complete the survey for the dog to which they were emotionally closest to, given that we specifically wished to study the effects of the dog who was most likely to be impacting the mental wellbeing of the owner through their relationship.

**Table 1:** The demographic characteristics of the dogs whose owners filled out the questionnaire.

Characteristics	Groups	Participants number (out of 1,693)	Percentage (%)
Dog’s sex	Male	861	51.1
	Female	824	48.9
	Missing	8	–
Dog’s neutered status	Yes	1,335	79.1
	No	352	20.9
	Missing	6	–
Dog’s size	Toy	54	3.2
	Small	474	28.1
	Medium	712	42.2
	Large	420	24.9
	Giant	28	1.7
	Missing	5	–
Dog’s age (in years)	<1	59	3.5
	1–4	661	39.3
	5–8	493	29.3
	9–12	334	19.8
	>12	107	6.4
	Unknown	29	1.7
	Missing	10	–
Dog’s weight status	Overweight	119	7.2
	Not overweight	1,533	92.8
	Missing	41	–
Dog’s location	Yard or garden	31	1.8
	Somewhere else	86	5.1
	Some rooms in the house	685	40.5
	All rooms in the house	889	52.6
	Missing	2	–
Dog’s primary caretaker	Participant	1,551	91.7
	Someone else	141	8.3
	Missing	1	–

The third block was named “Dog-owner relationship questions” and consisted of the Monash Dog-Owner Relationship Scale (MDORS; Howell et al., 2017), comprising three subsections: pet-owner interaction, perceived emotional closeness, and perceived costs. The fourth block, titled “Owner health-related questions,” had five parts. First, the participants were asked questions about their general health, including self-rating of their physical health, using the Patient-Reported Outcomes Measurement Information System (PROMIS) Global Items Scale (Ron et al., 2009). Second, they were asked the PROMIS item bank short forms: 8a for anxiety, 8b for depression, 4a for emotional support and 4a for companionship (Cella et al., 2007, 2010). Participants were then asked to answer 3 open-ended questions about how dog ownership improves their mental health, may make their mental health worse, and any challenges they may face in caring for their dog. Participants were also asked if they had ever been diagnosed with a mental health problem or if they had a physical disability. Finally, participants were asked about their physical activity levels using questions modified from the Dogs and Physical Activity Tool (Cutt et al., 2008), including time spent walking, cycling or jogging with their dog. The fifth and final block of the survey covered “Questions about yourself,” asking for demographic information (Table 1).

### Quantitative Analysis

The data were analyzed using the IBM SPSS Statistics software, version 24.0. Missing responses were not included.

### Dependent Variables

The MDORS questions (score 1–5 for each item) were used to create scores for the three subscales: pet-owner interaction, perceived emotional closeness and perceived costs (reverse scored); and a total score (see Supplementary Table 1). A higher score indicated a stronger relationship between the owner and their pet, i.e., high pet-owner interaction, high emotional closeness, or low perceived burden of owning the dog.

### Outcomes

Four PROMIS mental profile sum scores were calculated for each individual, which measured anxiety, depression, emotional support and companionship (see Supplementary Table 2). Scores were calculated so that higher score in anxiety, depression, emotional support and companionship scales indicated a poorer mental health status.

### Data Analysis

The outcomes were highly positively skewed and non-normal distributed. Linear regression was used to model the log10 of each psychological outcome against each dog–owner relationship subscale and relationship total score. Multivariable linear regression was performed in order to adjust for confounding variables of: age, gender, marital, work and educational statuses (Model 1); plus the minutes per week spent walking the dog (Model 2); plus the dog’s age and location in the house (Model 3); plus owner self-rated physical health status and mental health diagnosis (Model 4).

### Qualitative Analysis

The data submitted in response to the open questions was used to conduct thematic analysis (Braun and Clarke, 2006) using NVivo software, version 12. The responses to “describe any way you personally think owning a dog improves your mental health” was used to inform positive aspects of dog ownership; “Describe any way you personally think owning a dog makes your mental health worse” and “what are the biggest challenges you face in caring for your dog?” were used to generate themes on negative aspects of dog ownership. Line-by-line open coding was conducted by the first author (AM) in order to identify and categorize key themes; coding involved reading the participants’ answers and organizing similar responses into themes, then comparing and contrasting new responses into the same or further themes, or modifying previous themes as generated theory evolved. Coding was regularly discussed with author CW and emerging themes and their relationships to each other’s discussed also with TMG. Coding and categorization of the key themes was continued until theoretical saturation was reached and no new themes were emerging (Saunders et al., 2018).

## Results

### Sample Description

The survey had initially 2,437 responses: 41 were removed because they did not meet all the eligibility criteria; 701 responses were removed as mostly incomplete (especially regarding demographic information). Therefore, the total number of the final sample analyzed was 1,693 respondents (69.5%). Tables 1 and 2 summarize the socio-demographics of the sample. The typical profile of the individual that completed the questionnaire was a 25 to 45-year old female, overweight, married and living with one other person, childless, holding an undergraduate degree, employed, and living without any mental health diagnosis or any physical disability or chronic disease (Table 1). The typical profile of the dog had the participant as its primary caretaker, was neutered, medium size, 1–4 years old, normal weight and allowed access to all rooms of the house (Table 2).

**Table 2:** The demographic characteristics of the dog owner survey participants.

Characteristics	Groups	Participants number (out of 1,693)	Percentage (%)
Age (years)	18–24	212	12.6
	25–34	410	24.3
	35–44	350	20.7
	45–54	379	22.4
	55–64	233	13.8
	>65	105	6.2
	Missing	4	–
Gender	Male	155	9.2
	Female	1,519	90.3
	Other	8	0.5
	Missing	11	–
BMI (kg/m^2)	Overweight	706	53.2
	Not overweight	622	46.8
	Missing	365	–
People in household	1	255	15.1
	2	783	46.2
	3	314	18.5
	4	328	19.4
	>4	13	0.8
	Missing	0	–
Children under 16 years of age in household	Yes	314	18.7
	No	1,365	81.3
	Missing	14	–
Marital status	Married	704	42.1
	Living with a partner	401	24.0
	Divorced/Separated	141	8.4
	Widowed	25	1.5
	Never married	402	24.0
	Missing	20	–
Educational status	University Higher Degree (e.g., MSc, PhD)	313	19.4
	First degree level qualification including foundation degrees, Graduate membership of a professional institute, PGCE	507	31.4
	Diploma in Higher Education	219	13.6
	Teaching qualification (excluding PGCE)	27	1.7
	Nursing or other medical qualification not yet mentioned	63	3.9
	A Level	177	11.0
	Welsh Baccalaureate	0	0.0
	AS Level	16	1.0
	Higher Grade/Advanced Higher (Scotland)	24	1.5
	Certificate of sixth year studies	6	0.4
	GCSE/O Level	214	13.2
	GSE	33	2.0
	Standard/Ordinary (O) Grade / Lower (Scotland)	6	0.4
	Other school (including School leaving exam certificate or matriculation)	11	0.7
	None of the above	0	0.0
	Missing	77	-
Work status	Work for wage, payment or profit	1,274	76.7
	Unpaid work	48	2.9
	Retired	144	8.7
	Home duties	54	3.2
	Unemployed	142	8.5
	Missing	31	–
Mental health diagnosis	Yes	678	40.2
	No	960	56.9
	Do not wish to say	49	2.9
	Missing	6	-
Physical disability or chronic disease	Yes	350	20.8
	No	1,319	78.2
	Do not wish to say	17	1.0
	Missing	7	–

### Quantitative Findings

Participant responses are summarized in the Supplementary Materials: anxiety (Supplementary Table 3); depression (Supplementary Table 4); emotional support (Supplementary Table 5); companionship (Supplementary Table 6); pet-owner interaction (Supplementary Table 7); emotional closeness (Supplementary Table 8); perceived costs (Supplementary Table 9).

Table 3 presents the results of the adjusted and unadjusted analyses for anxiety, depression, companionship and emotional support outcomes. Green and red colors indicate an association which results in better or worse mental health of the owner, respectively. Model 1 results, adjusted for age, gender, marital, work and educational level are used to present the main findings.

**Table 3:** Linear regression examining whether pet-owner interaction, perceived emotional closeness, perceived costs and their sum are predictors of poorer mental health outcomes (logged).

Outcome	Variable	Unadjusted		Model 1		Model 2		Model 3		Model 4	
		B (95%CI)	P	B (95%CI)	P	B (95%CI)	P	B (95%CI)	P	B (95%CI)	P
Log anxiety	Total relationship	<0.001 (0.000–0.001)	0.311	<0.001 (−0.001–0.001)	0.868	<0.001 (−0.001–0.001)	0.855	<0.001 (−0.001–0.001)	0.781	<0.001 (−0.001–0.001)	0.741
	Pet-owner interaction subscale	0.001 (−0.001–0.003)	0.489	<0.001 (−0.002–0.002)	0.886	<0.001 (−0.002–0.003)	0.859	<0.001 (−0.002–0.003)	0.942	<0.001 (−0.003–0.002)	0.751
	Perceived emotional closeness subscale	0.006 (0.004–0.008)	<0.001	0.004 (0.003–0.006)	<0.001	0.004 (0.003–0.006)	<0.001	0.004 (0.002–0.006)	<0.001	0.004 (0.002–00.6)	<0.001
	Perceived costs subscale	-0.005 (−0.007 – −0.003)	<0.001	-0.005 (−0.007 – −0.003)	<0.001	-0.005 (−0.007 – −0.003)	<0.001	-0.005 (−0.007 – −0.003)	<0.001	−0.004 (−0.006 – −0.003)	<0.001
Log depression	Total relationship	0.001 (0.001–0.002)	0.002	0.001 (0.000–0.001)	0.127	<0.001 (0.000–0.000)	0.038	0.001 (0.000–0.002)	0.209	0.001 (0.000–0.002)	0.202
	Pet-owner interaction subscale	0.003 (0.001–0.005)	0.007	0.002 (0.000–0.004)	0.038	0.003 (0.000–0.005)	0.033	0.002 (0.000–0.005)	0.49	0.002 (0.000–0.004)	0.088
	Perceived emotional closeness subscale	0.008 (0.006–0.010)	<0.001	0.006 (0.004–0.008)	<0.001	0.006 (0.004–0.008)	<0.001	0.006 (0.004–0.008)	<0.001	0.005 (0.003–0.007)	<0.001
	Perceived costs subscale	−0.004 (−0.006 – −0.002)	<0.001	−0.005 (−0.006 – −0.003)	<0.001	−0.005 (−0.007 – −0.003)	<0.001	−0.005 (−0.007 – −0.003)	<0.001	−0.004 (−0.006 – −0.002)	<0.001
Log emotional support	Total relationship	−0.001 (−0.001–0.000)	0.150	−0.001 (−0.002–0.000)	0.020	−0.001 (−0.002–0.000)	0.017	−0.001 (−0.002–0.000)	0.018	−0.001 (−0.002–0.000)	0.012
	Pet-owner interaction subscale	−0.001 (−0.004–0.001)	0.267	−0.001 (−0.003–0.002)	0.510	−0.001 (−0.004–0.001)	0.304	−0.001 (−0.004–0.001)	0.336	−0.002 (−0.004–0.001)	0.223
	Perceived emotional closeness subscale	0.002 (0.000–0.004)	0.024	0.001 (−0.001–0.003)	0.292	0.001 (−0.001–0.003)	0.383	0.001 (−0.001–0.003)	0.462	<0.001 (−0.002–0.002)	0.663
	Perceived costs subscale	−0.004 (−0.006 – −0.002)	<0.001	−0.005 (−0.007–0.003)	<0.001	−0.005 (−0.007–0.003)	<0.001	−0.005 (−0.007–0.003)	<0.001	−0.005 (−0.007–0.003)	<0.001
Log companionship	Total relationship	−0.001 (−0.001–0.000)	0.160	−0.001 (−0.002–0.000)	0.001	−0.001 (−0.002 – −0.001)	0.001	-0.001 (−0.002 –0.001)	0.001	−0.002 (−0.002 – −0.001)	<0.001
	Pet-owner interaction subscale	−0.002 (−0.004–0.000)	0.035	−0.002 (−0.004–0.000)	0.054	−0.003 (−0.005–0.000)	0.026	−0.003 (−0.005–0.000)	0.033	−0.003 (−0.005 – −0.001)	0.016
	Perceived emotional closeness subscale	0.002 (0.000–0.004)	0.016	0.001 (−0.001–0.002)	0.464	0.001 (−0.001–0.002)	0.478	<0.001 (−0.001–0.002)	0.665	<0.001 (−0.002–0.002)	0.938
	Perceived costs subscale	−0.003 (−0.005 – −0.002)	<0.001	−0.005 (−0.007 – −0.003)	<0.001	−0.005 (−0.007 – −0.003)	<0.001	−0.006 (−0.007 – −0.004)	<0.001	−0.005 (−0.007 – −0.003)	<0.001

Green and red colors indicate an association which results in a better or worse mental health outcome of the owner, respectively.

CI, confidence interval.

Model 1: adjusted for: age, gender, marital status, work status and educational level.

Model 2: adjusted for: age, gender, marital status, work status, educational level and minutes of walking with the dog per week.

Model 3: adjusted for: age, gender, marital status, work status, educational level, minutes of walking with the dog per week, age of the dog and location of the dog.

Model 4: adjusted for: age, gender, marital status, work status, educational level, minutes of walking with the dog per week, age of the dog, location of the dog, physical health status, mental health problem diagnosis.

#### Anxiety

There was evidence of an association between high emotional closeness with their dog and higher (worse) anxiety score (Model 1 *B* = 0.004, 95%CI = 0.003–0.006, *p* < 0.001), and this association remained through all models. In contrast, there was also an association between high scores in the perceived costs of dog owning, i.e., perceived lower burden involved in dog ownership, and lower anxiety (Model 1 *B* = –0.005, 95%CI = –0.007 to −0.003, *p* < 0.001) and again this association remained through all models.

#### Depression

A higher total relationship score in the unadjusted analysis was associated with a higher (worse) depression score (*B* = 0.001, 95%CI = 0.001–0.002, *p* = 0.002), however after adjustment the association remained in Model 2 but not in Model 1 or any other model. Higher pet-owner interaction was associated with higher depression (Model 1 *B* = 0.002, 95%CI = 0.000–0.004, *p* = 0.038), and also remained in Model 2. Higher perceived emotional closeness was also associated with higher depression (Model 1 *B* = 0.006, 95%CI = 0.004–0.008, *p* < 0.001) and in further adjustments. However, a higher score in the perception of the costs of dog ownership, i.e., perceived lower burden involved in dog ownership, was associated with a lower (better) depression score (Model 1 *B* = –0.005, 95%CI = –0.006 to −0.003, *p* < 0.001), and remained with all adjustment.

#### Emotional Support

Even though there was no evidence of an association between the total relationship score and emotional support in the unadjusted analysis, for adjusted Model 1 a higher total relationship score was associated with lower (better) emotional support (*B* = –0.001, 95%CI = –0.002–0.000, *p* = 0.020), and this continued for Models 2–4. Higher perception of costs (i.e., lower burden of dog ownership) was associated with a lower (better) score in emotional support (Model 1 *B* = –0.005, 95%CI = –0.007 to −0.003, *p* < 0.001) and remained after further adjustment.

#### Companionship

The total relationship score was associated with a lower (better) companionship score in Model 1 (*B* = –0.001, 95%CI = –0.002 to 0.000, *p* = 0.001), and remained in Models 2–4. In the unadjusted analysis, a higher pet-owner interaction score was associated with a low companionship score, i.e., a healthier individual (*B* = –0.002, 95%CI = –0.004 to 0.000, *p =* –0.035), however this disappeared after adjustment in Model 1, but returned in Models 2, 3 and 4. Finally, higher perceived costs of dog ownership score (i.e., lower burden) was associated with a lower (better) companionship score, (Model 1 *B* = –0.005, 95%CI = –0.007 to −0.003, *p* < 0.001) and also remained with further adjustment.

#### Summary of Quantitative Findings

In summary, based upon Model 1 adjustments for age, gender, marital status, work status and educational level, higher (greater) pet-owner interaction was associated with a higher (worse) depression score (*B* = 0.002, 95%CI = 0.000 to 0.004, *p* = 0.038). Higher (greater) perceived emotional closeness was associated with higher (worse) anxiety (*B* = 0.004, 95%CI = 0.003 to 0.006, *p* < 0.001) and higher (worse) depression (*B* = 0.006, 95%CI = 0.004 to 0.008, *p* < 0.001). Higher score on perceived costs of dog ownership (lower burden) was associated with lower (better) scores for anxiety (*B* = –0.005, 95%CI = –0.007 to −0.003, *p* < 0.001), depression (*B* = –0.005, 95%CI = –0.006 to −0.003, *p* < 0.001), emotional support (*B* = –0.005, 95%CI = –0.007 to −0.003, *p* < 0.001), and companionship (*B* = –0.005, 95%CI = –0.007 to −0.003, *p* < 0.001). When the total MDORS scores were calculated, a closer dog–owner relationship was associated with lower (better) emotional support (*B* = –0.001, 95%CI = –0.002 to 0.000, *p* = 0.020) and companionship (*B* = –0.001, 95%CI = –0.002 to 0.000, *p* = 0.001).

### Qualitative Findings

**Figure 1:**
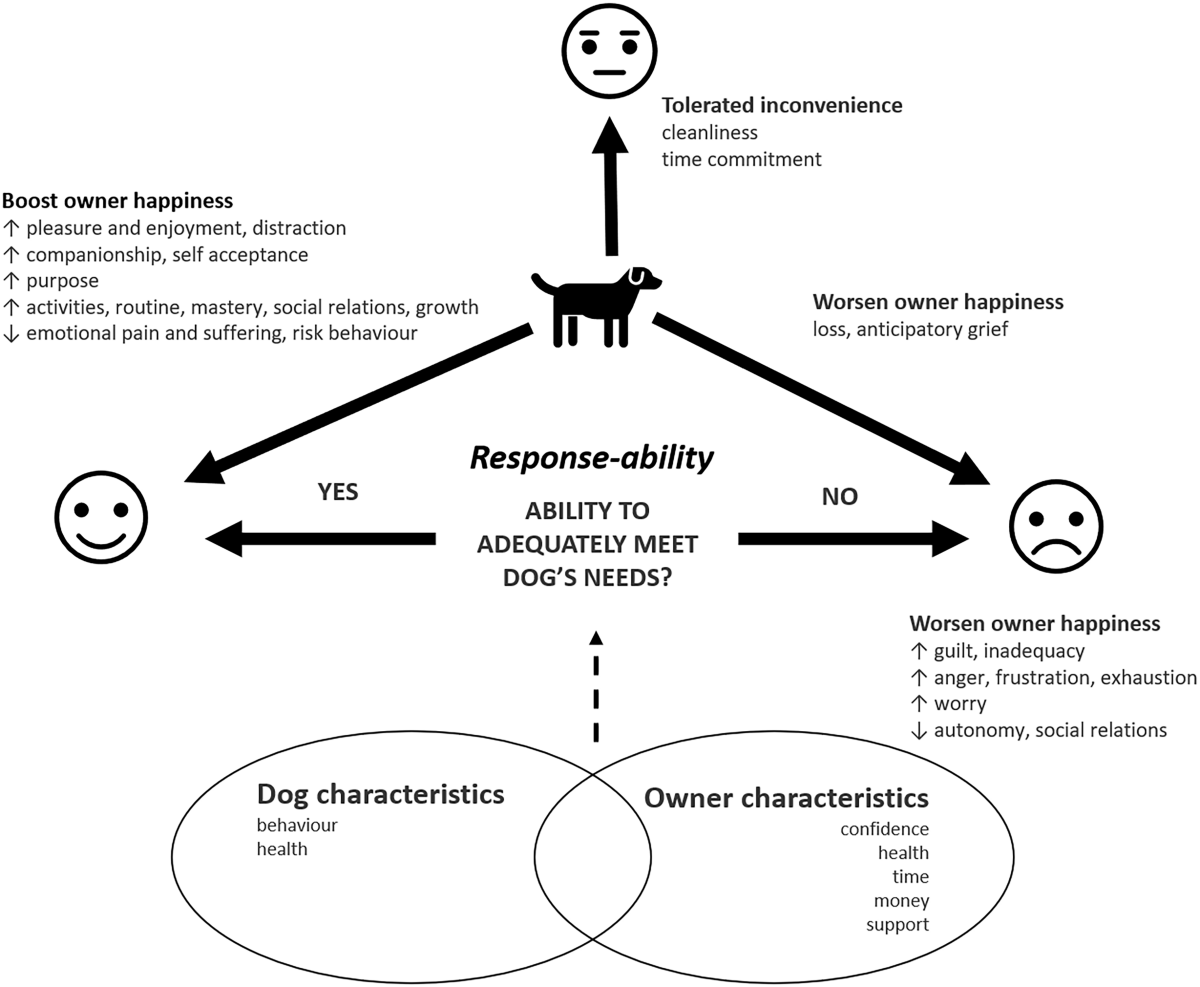
Thematic model of positive and negative impacts of dog ownership on owner wellbeing from qualitative analysis of open-text responses.

We found specific positive and negative aspect of owning pets which theoretically align with both hedonic and eudaimonic states (Figure 1).

#### How Dog Ownership Increases Owner Perceived Happiness

##### Dog Attributes Promote Pleasure and Enjoyment (↑ Hedonia)

Owners reported that their dogs made them laugh which provided simple pleasure and daily fun and entertainment:

“Keeps me entertained” (P45).

“My dog has taught me how to have fun again, something I did not think would happen again. She has taught me to live in the moment, and makes me laugh, truly laugh with joy” (P515).

“Always funny things happening when you own dogs, always! What’s not to like?” (P1484).

##### Dog Attributes Promote Feelings of Self-Acceptance (↑ Eudaimonia)

Dogs were described as devoted, loyal, and non-judgmental. Their companionship and presence made owners feel safe and valued:

“They are devoted, loyal” (P301).

“No matter what happens, they are the only thing that never judges you” (P720).

“Always here with unconditional love” (P198).

“Feeling like you matter is linked to your self-esteem and therefore mental health” (P1301).

##### Dogs and Their Activities Provide Purpose (↑ Eudaimonia)

Feeling a need to care for the dog motivated owners to get out of bed in the morning and to take better care of themselves:

“(My dog) gives me a reason to get up in the morning” (P1319).

“Doing things for him gives me a purpose and makes my life more fulfilled […] he means everything to me, and I am a better person for it” (P75).

“It’s a good feeling, to be needed. That this small life depends on me for most things. Looking after my dog means I take better care of me” (P681).

One owner stated that this purpose gave them more confidence and thus more autonomy too:

“He makes me feel less helpless, less annoying, more independent, more capable, more able to achieve” (P55).

Owning a dog was felt to be a privilege which they were grateful for, helped them to fulfill their potential and live more meaningfully and mindfully in the moment:

“Enables you to realise your potential for happiness and to live life to the fullest. Seeing how a dog can enjoy something seemingly mundane allows me to believe that happiness can be found anywhere” (P59).

In particular dogs provided owners with a purpose to get outdoors, where fresh air provided a new perspective:

“Fresh air and to walk, which I personally find really helps with stress and anxiety and getting a fresh perspective” (P5).

“Walking and fresh air leave me feeling mentally healthier, as does feeling like I’m making something else’s life enjoyable” (P1236).

“365 days of daily walks. Being outside on days you would not have bothered if there wasn’t a dog. Being part of the changing seasons” (P403).

##### Dog Activities Provide Mastery, Positive Relations and Personal Growth (↑ Eudaimonia)

The purpose and activities created by caring for a dog led to routines that were deemed helpful for structuring the day of the owner:

“Gives a routine to my day without which I would be faced with too many decisions about what to do and when” (P325).

Even when the weather was not conducive, owners reported mostly mastering this challenge and making the most of the opportunity:

“Sometimes it’s a groan in bad weather to go out but once out you meet people and smile and chat with other dog walkers, and when you get back you feel good” (P243).

Many owners described the roles that dogs played in promoting positive connections to others, mainly through dog walking:

“Having to go out to walk the dog twice a day makes you feel better, and you meet people and chat. Dog owners are almost always lovely” (P366).

Dogs also promoted owners to participate in activities that can contribute to the development of new skills and personal growth:

“My dog promotes a healthy lifestyle and encourages me to take part in hobbies and activities I would not take part without them” (P22).

##### Dog Attributes Lessen Owner Emotional Pain or Suffering (↑ Hedonia and Eudaimonia)

As a result of the innate nature of dogs, owners reported that they provided a positive distraction:

“[My dog] provides a model of positivity” (P205).

“[My dog] has helped me not to focus on my own problems” (P441).

Many owners described their dogs as intuitive and aware, providing comfort when they were unhappy:

“She instinctively knows when I’m unhappy and will come and sit alongside me” (P73).

“Cheers you up especially when you had a bad day” (P345).

This comfort appeared to help reduce mental health symptoms, with owners emphasizing the impacts on stress relief and depression:

“I have anxiety attacks nearly every day, sometimes more than once a day. Stroking my dog calms me down and stops my heart [from] racing. I’d be lost without [the dog]” (P117).

“I have depression and it was a lot worse before I had my dog. He cheers me up and gives me a reason to get up and out of the house” (P992).

“Someone to cuddle when I’m down” (P311).

“I can talk to them and share my thoughts with them, and this helps my mental health” (P390).

Dogs also helped ease the pain of loneliness:

“I never feel alone or lonely because I’m a dog owner” (P270).

Dogs also helped owners to deal with loss:

“I got my dog shortly after my husband was killed on his motorbike. She’s saved my life” (P324).

Finally, some owners reported that dogs helped them manage risk behavior, for example dealing with suicidal thoughts or self-harming, or relief from other symptoms:

“Honestly my dog was the main reason I stopped trying to kill myself and self-harming” (P281).

“Having my dog cured my lifelong fear of dogs and agoraphobia. She set me free” (P62).

“[My dog] saved my life, I was ready to end it a few years ago, but my family made me take her out for a walk and I came back in tears, I could not end my life because she would be out in the woods on her own […] I had to take her home and had to admit to my mental health issues” (P151).

#### How Dog Ownership May Decrease Owner Perceived Happiness

All participants had positive aspects of dog ownership to share; however, not all participants experienced negative aspects, with some reporting “nothing” at all.

##### Response-Ability Decreases Self-Acceptance, Environmental Mastery, Autonomy and Positive Relations (↓Eudaimonia)

*Meeting dog needs.* The main challenge related to the burden of responsibility of ownership was to do well by their dog by adequately meeting their needs, which involved negotiations and sacrifice on the owner’s part:

“Making sure [my dog] is fulfilled physically and mentally” (P264).

“Have to be committed and care for them always, they have to come first, and you sometimes have to miss things to ensure they are cared for” (P253).

A particular dog activity reported to pose challenges was walking:

“Walking every day (even when I feel exhausted or miserable)” (P103).

“The biggest and only challenge I have caring for my dog is walking her with other dogs. She’s nervous around other dogs. Bless her” (P340).

*Guilt.* Inability to meet dog’s needs in the way owners wanted (in a sense, response-inability) led to feelings of inadequacy and guilt, which in turn could worsen owner’s confidence and mental health:

“Feeling like a failure when struggling to give the pet the attention it needs all the time” (P67).

“It can increase worry, anxiety, feelings of guilt. Am I training him properly? Is he OK when he’s left alone? Am I feeding him the best food? Feeling guilty when he’s not been walked enough because of [my] low mood” (P196).

“When I am too anxious to go out on a dog walk, I feel guilty and useless that I cannot do the right thing for my dogs” (P411).

*Worry.* Participants expressed worries about their dogs getting sick, of themselves dying first, and/or of their dog getting stolen:

“Biggest worry is worrying about them getting ill or injured” (P112).

“Worry about what would happen to the dog if we were not here for her” (P201).

“Making sure she’s safe. So many issues these days with dogs being stolen” (P424).

The burden of responsibility felt and perceived ability to adequately meet dog’s needs (thus response-ability) appeared to be dependent on both owner characteristics and dog characteristics.

###### Owner Characteristics

*Confidence.* Some owners felt overwhelmed by the responsibility of deciding how to best provide for their dogs, given all the conflicting information out there, and considering issues regarding agency and that pets are unable to speak or decide for themselves:

“Knowing how best to train them for their happiness and health” (P214).

“Having to make decisions on their behalf when it comes to their healthcare” (P203).

*Time.* Balancing pet care with work or other family responsibilities could also be challenging:

“I wish I had more time to train him properly, but I have to juggle work and childcare, I sometimes feel guilty about this” (P827).

*Money.* Costs of pet care were frequently mentioned as a challenging factor that could influence ability to adequately meet dog’s needs:

“Financial issues such as medications or unexpected circumstances” (P70).

“Affording to give her the life she deserves” (P1679).

*Health.* Also shaping ability to adequately meet dog’s needs was owner’s physical and mental health:

“Physically being capable to meet his demands, though my carers help in that aspect” (P55).

“Sometimes when I am more unwell than usual, when my chronic pain and fatigue flare up or if I get a virus, then I can feel mentally worse as I know she needs to go out for walks or that she wants to play but I struggle to do it. I can then feel very low and helpless because I am upset or annoyed at my ability to provide stimulation and fun for her” (P300).

“Owning a dog comes with difficulties, e.g., dealing with illness, which due to the nature of my mental health difficulties can be very difficult for me, but I accept this is the case” (P74).

*Support.* Owners relied on support from others to help care for their dog and without access shared challenges. Owners worried about leaving the dog, and who they could trust their dog with, which limited ability to travel or socialize (autonomy and positive relations):

“Sometimes when I am depressed, I struggle to deal with him. He is incredibly energetic, and I do not have the energy for it but feel guilty for not interacting with him as much, so it makes me anxious and overwhelmed. Luckily, I live with my mum and she helps me take care of him when I cannot” (P1404).

“Getting reliable cover when I am unable to walk my dogs can be an issue” (P1025).

“Holidays as we will not leave him in kennels as he finds it too stressful and so do we, so we do not go away unless family can care for him” (P782).

####### Dog Characteristics

*Health.* Sometimes the health of the dog presented challenges in terms of the burden of responsibility to meet the dog’s needs:

“The dog I chose for the survey has IBD and a spinal injury. Because of this, she needs careful exercise, special feeding and protection from stress. This is hard work on a daily basis” (P400).

“If my dog is unwell or if I am worried about her health then it negatively affects my mental health” (P1444).

*Behavior.* Aggression, barking, separation anxiety, phobias (mainly noise-related), hyperactivity and other challenging behaviors were also highlighted as problematic:

“Coping with her aggression and anxiety issues” (P54).

“Makes me scared or worried when she barks in the middle of the night” (P1403).

“Sometimes when I am depressed, I struggle to deal with him. He is incredibly energetic, and I do not have the energy for it but feel guilty for not interacting with him as much, so it makes me anxious and overwhelmed. Luckily, I live with my mum and she helps me take care of him when I cannot” (P1404).

“With her being a puppy it is the constant cleaning up and disobedience at times which makes me feel worn out and worthless when she does not listen” (P138).

As well as creating feelings of guilt, inadequacy and worry, dog behaviors also led to feelings of anger, frustration and exhaustion:

“Frustrating if they make a mess” (P470).

“Sometimes they can drive you a little crazy or do something to annoy you” (P1338).

“I become agitated, angry and very overwhelmed with my small dog initiating barking at things, when my big dog will sometimes join in. I feel stressed then and often wonder whether my small dog is good for my mental health. Although I could never part with her, I love her too much” (P1364).

“The breed requires a lot of attention and he does not really care if I am unwell, he still wants to use up his energy. It can be exhausting at times!” (P1098).

In particular, behavior characteristics of the dog could impact the ability to leave them alone at home, contributing to a loss of spontaneity for the owner:

“She shreds paper and plastic so makes enormous mess and cannot be left with anything important alone. If she would grow out of this she would have more access to the house and I would enjoy her more. A bit exhausting!” (P273).

“My dog has severe separation anxiety and cannot be left at home unattended” (P139).

“I worry a lot about [my dog] …My time outside the home can be limited because I will not leave her alone for long periods. Can affect my social life at times” (P283).

##### Anticipatory Grief (↓ Hedonia)

Finally, owners worried in advance about how they would cope in future when their dog died, impacting their pleasurable emotions in the current moment:

“The only thing is that I will be devastated when I lose him and might get very depressed” (P33).

##### Tolerated Inconvenience

There were some aspects of dog ownership that were recognized as negative, but owners reflected that they tolerated them more as an inconvenience that was worth it, typically cleanliness issues such as mud and hair, or the time commitment involved:

“I suffer from allergies and asthma so obviously the battle to keep the house clean and allergen free can be tough at times as a dog owner especially as my dog sheds quite heavily. […] wet, muddy paws all over the floor can also make me feel a bit stressed so I find myself constantly trying to keep on top of that. I think the benefits of dog ownership far outweigh the negatives though” (P1424).

“My dog does not like to be left alone so I have to take her pretty much everywhere with me! It can be a little tricky but me and my partner manage and she’s worth it!” (P899).

#### Summary of Qualitative Findings

Dogs appeared to contribute to hedonic wellbeing in two important ways: by promoting pleasure and enjoyment and by lessening pain and suffering (e.g., providing comfort and distraction, helping to ease the pain of loneliness and loss, and reducing engagement in risky behavior). Dogs also contributed to aspects of eudemonic wellbeing, specifically through providing meaning and purpose, and encouraging positive relations with others and activities that can lead to personal growth and feelings of mastery. Their characteristics as non-judgmental and loving contributed to feelings of positive self-worth. However, the responsibility of caring for a dog and the perception of whether their needs were being met (response-ability) carried a large burden, in particular, where dog behavioral and physical health characteristics were problematic or the owner’s physical or mental health needs prevented them from caring for their dog in their perceived ideal way. The burden of these responsibilities may contribute to owners being unable to control their environment, accept themselves fully, be as autonomous as they would like, or establish good relations with others. Due to their shorter lifespans, owners also anticipated the eventual loss of their dog as being difficult to deal with.

## Discussion

### Summary of Findings

The aim of this paper was to investigate the association between owners’ relationship with their dogs and impacts on owner mental wellbeing, using both quantitative and qualitative approaches. Our findings appear at first somewhat in conflict, in that owners who have close relationships with their dogs feel more emotionally supported and have a stronger sense of companionship, but this does not appear to result in lower scores in anxiety and depression. The qualitative findings elucidate why this may be the case. Dogs increase their owners’ hedonic and eudaimonic wellbeing by helping owners enjoy themselves, and feel accepted, purposeful and more able to achieve. The activities done with dogs in particular act as motivators for more positive interpersonal relationships, give purpose and structure in their lives, and help their owners to “grow.” Dogs show support and love to their owners, and they make them feel valued through the responsibilities of caregiving; particularly appreciated by participants who mentioned that they have a mental health diagnosis. However, managing dogs’ physical health, behavioral issues and performing some dog-related activities can be a burden at times, negatively affecting sense of autonomy and opportunities to meet others. Further, worrying about their future can create anxiety. This caregiver burden can lead to negative feelings such as frustration, exhaustion, and in particular if owners perceive they are not fulfilling the dog’s needs, inadequacy and guilt. Therefore, depending on the owner, the dog, and their unique relationship, pet ownership can either positively or negatively affect owner mental wellbeing. However, crucially, dogs also help owners manage other aspects of their negative emotions and/or mental health difficulties like anxiety, depression and suicidal thoughts. This suggests that owners may seek out emotional support from their dogs in order to assist with managing their mental wellbeing, but this does not come without some burden of its own that warrants further investigation.

### Comparison With Previous Literature

Our contradictory findings regarding anxiety and depression and different scales of the dog–owner relationship corroborate the existing literature, as some studies find a positive relationship between pet ownership/dog–owner relationships and mental wellbeing (Zimolag and Krupa, 2009; González RamÍrez and Landero Hernández, 2011; Bakerjian, 2014; Wheeler and Faulkner, 2015; Brooks et al., 2016; Mota Pereira and Fonte, 2018; González-Ramírez et al., 2019; Liu et al., 2019; Powell et al., 2019; Hawkins et al., 2021), others a negative relationship (Parslow et al., 2005; Peacock et al., 2012; Bradley and Bennett, 2015; Enmarker et al., 2015; Mueller et al., 2018; Sharpley et al., 2020) and others none (Fraser et al., 2020; Le Roux and Wright, 2020). In our research, the perceived costs subscale consistently showed a lower perceived burden was positively associated with all mental health outcomes, including anxiety and depression. This is supported by our qualitative analysis, where our findings are in accordance with the paper of Barcelos et al. (2020), suggesting a mostly positive relationship which, however, comes with costs to owner mental wellbeing, particularly regarding the burden of responsibility and caregiving.

Our findings also support others who found that dogs can help in managing and alleviating mental health conditions, especially during times of crisis (Brooks et al., 2018). Even though our quantitative data showed that stronger emotional closeness with the dog was associated with poorer anxiety and depression, this is likely to be in part due to reverse causality and seeking out support from dogs (a form of co-dependency), as our qualitative research showed that the presence of the dog sometimes lessened mental health symptoms, such as suicidal thoughts or self-harming. Dog ownership may be valued for its protective effects, especially in at risk populations, such as LGBTQ+ emerging adults (Mcdonald et al., 2021), adults with a diagnosis of autism (Barcelos et al., 2021), or older adults who are at higher risk of fatal first suicidal attempts (Figueiredo et al., 2015; Young et al., 2020), and this requires further investigation. There are perhaps parallels to consider regarding the caregiving responsibility of parenthood and suggested impacts on lower suicide rates in adults with children (Dehara et al., 2021).

In some circumstances, owners wrote that their dogs supported them in extremely difficult situations, e.g., loss of a loved one. Previous research has found that dog ownership may promote resilience in the face of adversity (Applebaum et al., 2021) and decreases loneliness (Staats et al., 2008; Powell et al., 2019; Hui Gan et al., 2020), in particular in individuals with low human social support (Antonacopoulos and Pychyl, 2010). The emotional support provided by dogs appears to be of particular importance, and echoes other findings that a closer pet-owner relationship is associated with a more socially supported individual (Joseph et al., 2019). The provision of direct and indirect emotional support could be particularly important for those with mental health difficulties (Brooks et al., 2018), or marginalized populations, such as racialized minority populations, sexual and gender minority populations (Applebaum et al., 2021).

In addition to providing support in managing negative emotions, dogs also promoted positive emotions, and thus improved the owners’ hedonic and eudaimonic wellbeing in this manner. Owners reported increased happiness, mindful presence, gratefulness, and confidence from their interaction with their dogs, and enjoyed themselves around them, especially when dogs do something the owners perceive as amusing, similar to reported previously (Westgarth et al., 2017; Owczarczak-Garstecka et al., 2021). Attributes of dogs that led to positive mental wellbeing effects included the concept of the dog being loyal, non-judgmental and showing empathy and unconditional love. Communication between the dog and the owner using non-verbal language “two-way” and contributes to the owner feeling valued, heard and understood (Maharaj and Haney, 2015) and engenders the sense of responsibility for the pet as “kin” (Westgarth et al., 2019). This leads to caregiving behavior which further improved eudaimonic wellbeing through providing motivation, purpose and a routine. Owners reported that through dog-related activities, mainly dog walking, dogs were a motivator for them to get of the house, meet new people and even uptake new dog-related hobbies. Even though some owners struggled to find the motivation to walk with their dogs, especially when experiencing depressive symptoms, most of them commented that once they did go outside, it had many positive impacts that they were thankful for. Dog walking has previously been shown to generate feelings of happiness, through sharing of vicarious pleasure, stress relief, and promoting social connection (Westgarth et al., 2017b) and dogs are known to increase social connections within communities (Wood et al., 2007; Graham and Glover, 2014). However, unlike suggested by Barcelos et al. (2020) our owners tended to not volunteer information that suggested that greater autonomy was created as a result of owning a dog, rather than a particular downside of caregiving responsibilities is reduced autonomy by preventing owners from being able to pursue other interests or relationships, similar to that found by others (Graham et al., 2019).

Indeed, positive effects of dog ownership appeared to be conditional to particular contexts, in particular their experience of behavioral problems which were frustrating and exhausting. Other research concurs that owner and animal welfare are strongly linked. Owners that have well behaved pets when left alone were happier whereas owners who witnessed separation-related anxiety problems in their dogs were stressed (González-Ramírez et al., 2018). Unpicking directions of causality is difficult; however some researchers suggests that although human and dog long-term anxiety are strongly correlated, it is dogs mirroring their owners’ stress levels (Sundman et al., 2019). Dogs’ physical illnesses and behavioral problems, such as aggression, barking, anxiety, phobias and training problems, can affect the owners’ psychological wellbeing as they feel less autonomous, and have a lower regard for self and it further restricts their management of everyday affairs and social life.

Responsibility for caring for a dog also comes with wider burdens. Our findings agree with the results of other studies, that dog walking is overall pleasurable but also able to generate negative feelings when perceived as a chore and that guilt and the dog’s needs are the primary motivator for the walk (Westgarth et al., 2014, 2019, 2021). While perceived ability to meet dog’s needs has been identified previously to negatively impact owner wellbeing (Barcelos et al., 2020), our study more deeply elucidates how this is underpinned and influenced by characteristics of the dog and the owner. For example, owners’ physical or mental limitations, restrictions such as finance, caregiving knowledge, time, work and social support available were identified as impacting perceived ability to caregive. Most owners consider these stressful challenges to negotiate, as identified in older adults where responsibilities such as costs and cleaning can compromise their wellbeing (While, 2017). It is also interesting to point out that some owners thought that the lack of agency of the dog to control its own life and responsibility of deciding on the dog’s behalf compounded this stress and feelings of inadequacy. When individuals worry about something negative that might happen in future, it can further increase their anxiety in the present (Schubert et al., 2020), and we found that owners often worried about what might happen to their dog or what would happen to the dog if they die first. They also suffered anticipatory grief (Spitznagel et al., 2021) about how they would cope with the loss of their dog. Even though the caregiving responsibilities are stressful, it is interesting to note that caregiving for a severely ill animal has been suggested to create less burden and more positive attitude compared to caregiving for a human family member (Karysa et al., 2018).

These burdens added to the other noted instances of feeling exhausted, annoyed, challenged, frustrated, and overwhelmed, may suggest that the added responsibility of caring for a dog can be challenging for owner mental wellbeing and acquiring a dog may not always be a suitable choice. Further, our multivariable regression analyses showed that owner characteristics, such as age, marital and work statuses and whether there was a mental health problem diagnosis, were independently associated with anxiety, depression, emotional support and companionship. This confirms that that a close relationship with a dog is not the only factor affecting the mental health of the owner, and these also need to be taken into account when considering whether dog ownership is a suitable course of action.

In our study, some owners explicitly stated that there was “nothing” negative in owning a dog. and many highlighted that dog ownership “is worth it,” i.e., the positives outweigh any negatives, as observed in previous research (Westgarth et al., 2019). Therefore, although there may be difficulties that dog ownership can bring, which may be particularly challenging to someone already living with a mental health diagnosis, overall, owning a dog appears to provide invaluable benefits to the owner. Our findings suggest that the key to a healthy dog–owner relationship that supports owner mental wellbeing is ensuring the right supports are in place, including but not limited to: affordable veterinary care and dog training, walking, and boarding services, access to pet-friendly housing and dog-supportive environments, and mental health support for people who may be struggling with anticipatory grief or who may have recently lost a pet, particularly for those with limited social support.

### Strengths and Limitations

A strength of this study is its large sample, especially for the qualitative aspects, but a limitation is that it is a cross-sectional study with self-reported replies rather than objective measures of mental wellbeing. The qualitative responses are inductive rather than deductive, but were limited by being relatively short replies rather than in-depth conversation. It is also a convenience sample as people completed the questionnaire at their discretion in response to a social media advert. Moreover, it is an almost female-only sample, as is the majority of human-animal interaction research (Rodriguez et al., 2021). Tower and Nokota suggested that women benefit more from a pet compared to men (Tower and Nokota, 2006), suggesting that our findings may not necessarily fully apply in the male owner context, and further research into this is required. Further, a relatively high proportion (40%) felt that they had a mental health diagnosis, which may reflect some recruitment bias but is a useful sample for our research questions. Looking into disadvantaged populations also needs further investigation, as mental health difficulties may be even more prominent and the human-animal bond more complex (Applebaum et al., 2021). Additionally, it should be pointed out that the scales taken from the PROMIS for emotional support and companionship did not state clearly if they concerned humans only and thus participants may have varied in their interpretations of these questions.

Finally, reverse causation in a cross-sectional study should be taken into consideration as the directionality of the relationship is difficult to infer. However, the novel mixed-method nature of this particular study is a strength that helps elucidate how causality may be occurring. It may be that people who are closer to their dogs are more inclined to develop mental health difficulties, or those who already have depression or anxiety are more likely to get a dog in order to manage its symptoms and/or are more inclined toward becoming more strongly emotionally attached to their dogs. Our qualitative findings suggest that the latter explanations are certainly playing a part. Further, even though some participants mentioned that caring for a dog is sometimes stressful and overwhelming, especially on their “bad days,” no participant explicitly said that they were mentally healthy previously and getting a dog created a mental health problem; in fact, the opposite was often stated.

### Conclusion

It is often believed that dogs can bring many mental health benefits to their owners, and hypothesized that the closer the relationship, the greater those benefits, but our findings somewhat contradict these assumptions. It was found that a closer relationship is associated with higher feelings of emotional support and companionship, but poorer levels of anxiety and depression. Yet many mental health benefits, and mechanisms of increasing experiences of hedonia and eudaimonia, were described due to interactions with dogs. Dogs were also mentioned as a useful aid for dealing with mental health symptoms, such as suicidal thoughts. Hence, we suggest that a close dog–owner relationship may be a feasible strategy to help people cope during mentally challenging times, but they are not a panacea in terms of prevention or treatment for depression or anxiety. It must be noted that every person is different, every dog is different, and the dog–owner relationship comes with many responsibilities and challenges. Longitudinal research is needed to further investigate for whom, and in what context, dog ownership is a feasible strategy to contribute to positive wellbeing, and how to ensure the dog’s health and behavioral welfare is also high, not least because without this, our findings show that dog ownership can become a challenging burden. The perceived costs subscale demonstrated the most consistent associations with wellbeing outcomes and thus it may the most useful representation of the dog–owner relationship in regards to impact on owner wellbeing. A lower perceived burden was beneficially associated with all mental health outcomes, including lower anxiety and depression, and also was found to be important in our qualitative explorations, and so it is important for future research and practical interventions to address issues that lead to a sense of burden created by owning and caring for a dog.

## Data Availability Statement

The raw data supporting the conclusions of this article will be made available by the authors, without undue reservation.

## Ethics Statement

The studies involving human participants were reviewed and approved by University of Liverpool Veterinary Ethics Committee. The patients/participants provided their written informed consent to participate in this study. The animal study was reviewed and approved by University of Liverpool Veterinary Ethics Committee. Written informed consent was obtained from the owners for the participation of their animals in this study.

## Author Contributions

CW devised and designed the study, assisted qualitative and quantitative data analysis, and drafted the manuscript. AM designed the data collection, collected the data, performed all analysis, and drafted the manuscript. TG assisted with qualitative data analysis and supported drafting of the manuscript. MO’H and RP assisted with study design, advised on quantitative data analysis, and commented on the manuscript. All authors contributed to the article and approved the submitted version.

## Funding

AM summer studentship funded by Wellcome Trust Institutional Strategic Support Fund awarded to University of Liverpool.

## Conflict of Interest

The authors declare that the research was conducted in the absence of any commercial or financial relationships that could be construed as a potential conflict of interest.

## Publisher’s Note

All claims expressed in this article are solely those of the authors and do not necessarily represent those of their affiliated organizations, or those of the publisher, the editors and the reviewers. Any product that may be evaluated in this article, or claim that may be made by its manufacturer, is not guaranteed or endorsed by the publisher.

## References

[ref1] Antonacopoulos N. M. D. Pychyl T. A. (2010). An examination of the potential role of pet ownership, human social support and pet attachment in the psychological health of individuals living alone. Anthrozoös 23, 37–54. doi: 10.2752/175303710X12627079939143

[ref3] Applebaum J. W. Maclean E. L. Mcdonald S. E. (2021). Love, fear, and the human-animal bond: On adversity and multispecies relationships. Comprehensive Psychoneuroendocrinology 7:100071. doi: 10.1016/j.cpnec.2021.100071, PMID: 34485952 PMC8415490

[ref4] Bakerjian D. (2014). Pets impact on quality of life, a case study. Geriatr. Nurs. (New York, N.Y.) 35, 160–163. doi: 10.1016/j.gerinurse.2014.02.009, PMID: 24829964

[ref5] Barcelos A. M. Kargas N. Maltby J. Hall S. Mills D. S. (2020). A framework for understanding how activities associated with dog ownership relate to human well-being. Sci. Rep. 10:11363. doi: 10.1038/s41598-020-68446-9, PMID: 32647301 PMC7347561

[ref6] Barcelos A. M. Kargas N. Packham C. Mills D. S. (2021). Understanding the impact of dog ownership on autistic adults: implications for mental health and suicide prevention. Sci. Rep. 11:23655. doi: 10.1038/s41598-021-02504-8, PMID: 34880277 PMC8655007

[ref7] Barker S. B. Wolen A. R. (2008). The benefits of human-companion animal interaction: a review. J. Vet. Med. Educ. 35, 487–495. doi: 10.3138/jvme.35.4.487, PMID: 19228898

[ref8] Bradley L. Bennett P. C. (2015). Companion-animals’ effectiveness in managing chronic pain in adult community members. Anthrozoös 28, 635–647. doi: 10.1080/08927936.2015.1070006

[ref9] Braun V. Clarke V. (2006). Using thematic analysis in psychology. Qual. Res. Psychol. 3, 77–101. doi: 10.1191/1478088706qp063oa

[ref10] Brooks H. L. Rushton K. Lovell K. Bee P. Walker L. Grant L.. (2018). The power of support from companion animals for people living with mental health problems: a systematic review and narrative synthesis of the evidence. BioMed Central. 18:31. doi: 10.1186/s12888-018-1613-2, PMID: 29402247 PMC5800290

[ref11] Brooks H. Rushton K. Walker S. Lovell K. Rogers A. (2016). Ontological security and connectivity provided by pets: a study in the self-management of the everyday lives of people diagnosed with a long-term mental health condition. BMC Psychiatry 16, 1–12. doi: 10.1186/s12888-016-1111-3 27931210 PMC5146817

[ref12] Cella D. Riley W. Stone A. Rothrock N. Reeve B. Yount S.. (2010). The patient-reported outcomes measurement information system (PROMIS) developed and tested its first wave of adult self-reported health outcome item banks: 2005–2008. J. Clin. Epidemiol. 63, 1179–1194. doi: 10.1016/j.jclinepi.2010.04.011, PMID: 20685078 PMC2965562

[ref13] Cella D. Yount S. Rothrock N. Gershon R. Cook K. Reeve B.. (2007). The patient-reported outcomes measurement information system (PROMIS): Progress of an NIH roadmap cooperative group during its first two years. Med. Care 45, S3–S11. doi: 10.1097/01.mlr.0000258615.42478.55, PMID: 17443116 PMC2829758

[ref14] Cutt H. E. Giles-Corti B. Knuiman M. W. Pikora T. J. (2008). Physical activity behavior of dog owners: development and reliability of the dogs and physical activity (DAPA) tool. J. Phys. Act. Health 5, S73–S89. doi: 10.1123/jpah.5.s1.s73, PMID: 18364529

[ref15] Dehara M. Wells M. B. Sjöqvist H. Kosidou K. Dalman C. Sörberg Wallin A. (2021). Parenthood is associated with lower suicide risk: a register-based cohort study of 1.5 million swedes. Acta Psychiatr. Scand. 143, 206–215. doi: 10.1111/acps.13240, PMID: 33011972 PMC7983926

[ref16] Enmarker I. Hellzén O. Ekker K. Berg A.-G. T. (2015). Depression in older cat and dog owners: the Nord-Trøndelag Health Study (HUNT)-3. Aging Ment. Health 19, 347–352. doi: 10.1080/13607863.2014.933310 24990174

[ref17] Figueiredo A. E. B. Silva R. M. D. Vieira L. J. E. S. Mangas R. M. D. N. Sousa G. S. D. Freitas J. S.. (2015). É possível superar ideações e tentativas de suicídio? Um estudo sobre idosos/is it possible to overcome suicidal ideation and suicide attempts? A study of the elderly. Cien. Saude Colet. 20, 1711–1719. doi: 10.1590/1413-81232015206.02102015, PMID: 26060949

[ref19] Fraser G. Huang Y. S. Robinson K. Wilson M. S. Bulbulia J. Sibley C. G. (2020). New Zealand pet owners' demographic characteristics, personality, and health and wellbeing: more than just a fluff piece. Anthrozoös 33, 561–578. doi: 10.1080/08927936.2020.1771060

[ref20] Gilbey A. Tani K. (2015). Companion animals and loneliness: a systematic review of quantitative studies. Anthrozoös 28, 181–197. doi: 10.1080/08927936.2015.11435396

[ref21] González Ramírez M. T. Landero Hernández R. (2011). Diferencias en Estrés Percibido, Salud mental y Física de acuerdo al Tipo de Relación Humano-Perro/differences in perceived stress, mental health, and physical health according to types of human-pet dog relationships. Revista Colombiana de Psicología 20, 75–86.

[ref22] González-Ramírez M. T. Quezada-Berumen L. D. C. Landero-Hernández R. (2019). FElicidad Subjetiva Después De Vivir Un Evento Traumático En Personas Con y Sin Animales De Compañía. Acción Psicológica 16, 91–104. doi: 10.5944/ap.16.1.23440

[ref23] González-Ramírez M. T. Vanegas-Farfano M. Landero-Hernández R. (2018). Differences in stress and happiness between owners who perceive their dogs as well behaved or poorly behaved when they are left alone. J. Vet. Behav. 28, 1–5. doi: 10.1016/j.jveb.2018.07.010

[ref24] Graham T. M. Glover T. D. (2014). On the fence: dog parks in the (un)leashing of community and social capital. Leis. Sci. 36, 217–234. doi: 10.1080/01490400.2014.888020

[ref25] Graham T. M. Milaney K. J. Adams C. L. Rock M. J. (2019). Are Millennials really picking pets over people? Taking a closer look at dog ownership in emerging adulthood. Can. J. Fam. Youth 11, 202–227. doi: 10.29173/cjfy29454

[ref26] Hawkins R. D. Hawkins E. L. Tip L. (2021). 'I Can't Give Up When I Have Them to Care for': People's Experiences of Pets and Their Mental Health. Great Britain. Taylor & Francis.

[ref27] Howell T. J. Bowen J. Fatjó J. Calvo P. Holloway A. Bennett P. C. (2017). Development of the cat-owner relationship scale (CORS). Behav. Process. 141, 305–315. doi: 10.1016/j.beproc.2017.02.024, PMID: 28279780

[ref28] Hui Gan G. Z. Keesing S. Netto J. A. Hill A. M. Yeung P. (2020). Pet ownership and its influence on mental health in older adults. Aging Ment. Health 24, 1605–1612. doi: 10.1080/13607863.2019.1633620, PMID: 31242754

[ref29] Joseph N. Chandramohan A. K. D'souza A. L. Basavanna S. C. Hariram S. Nayak A. H. (2019). Assessment of pet attachment and its relationship with stress and social support among residents in Mangalore city of South India. J. Vet. Behav. 34, 1–6. doi: 10.1016/j.jveb.2019.06.009

[ref30] Karysa B. Rachel G. Geoffrey T. Kimberly C. Olivia H. Mark D. C.. (2018). Caregiving for a companion animal compared to a family member: burden and positive experiences in caregivers. Front. Vet. Sci. 5:325. doi: 10.3389/fvets.2018.00325 30619903 PMC6308119

[ref32] Le Roux M. C. Wright S. (2020). The relationship between pet attachment, life satisfaction, and perceived stress: results from a south African online survey. Anthrozoös 33, 371–385. doi: 10.1080/08927936.2020.1746525

[ref33] Liu S. Powell L. Chia D. Bauman A. E. Stamatakis E. Russ T. C.. (2019). Is dog ownership associated with mental health? A population study of 68,362 adults living in England. Anthrozoös 32, 729–739. doi: 10.1080/08927936.2019.1673033

[ref34] Maharaj N. Haney C. J. (2015). A qualitative investigation of the significance of companion dogs. West. J. Nurs. Res. 37, 1175–1193. doi: 10.1177/0193945914545176, PMID: 25092206

[ref35] Mcdonald S. E. Matijczak A. Nicotera N. Applebaum J. W. Kremer L. Natoli G.. (2021). “He was like, my ride or die”: sexual and gender minority emerging adults’ perspectives on living With pets During the transition to adulthood. Emerg. Adulthood 216769682110253. doi: 10.1177/21676968211025340

[ref36] Mota Pereira J. Fonte D. (2018). Pets enhance antidepressant pharmacotherapy effects in patients with treatment resistant major depressive disorder. J. Psychiatr. Res. 104, 108–113. doi: 10.1016/j.jpsychires.2018.07.004, PMID: 30025233

[ref37] Mueller M. K. Gee N. R. Bures R. M. (2018). Human-animal interaction as a social determinant of health: descriptive findings from the health and retirement study. BMC Public Health 18:305. doi: 10.1186/s12889-018-5188-0, PMID: 29519232 PMC5844080

[ref38] Nice. (2019). NICEimpact mental health [Online]. Available at: https://www.nice.org.uk/Media/Default/About/what-we-do/Into-practice/measuring-uptake/NICEimpact-mental-health.pdf. (Accessed August 13, 2020).

[ref39] Owczarczak-Garstecka S. C. Graham T. M. Archer D. C. Westgarth C. (2021). Dog walking before and during the COVID-19 pandemic lockdown: experiences of UK dog owners. Int. J. Environ. Res. Public Health 18:6315. doi: 10.3390/ijerph18126315, PMID: 34200926 PMC8296116

[ref40] Oxford Economics (2016). Added Value: Mental Health as a Workplace Asset Oxford Economics. Available at: https://www.oxfordeconomics.com/resource/added-value-mental-health-as-a-workplace-asset/

[ref41] Parslow R. A. Jorm A. F. Christensen H. Rodgers B. Jacomb P. (2005). Pet ownership and health in older adults: findings from a survey of 2,551 community-based australians aged 60-64. Gerontology (Basel) 51, 40–47. doi: 10.1159/000081433, PMID: 15591755

[ref42] Peacock J. Chur-Hansen A. Winefield H. (2012). Mental health implications of human attachment to companion animals. J. Clin. Psychol. 68, 292–303. doi: 10.1002/jclp.20866, PMID: 22307948

[ref43] Pope C. Mays N. (1995). Reaching The parts other methods cannot reach: an introduction to qualitative methods In health And health services research. BMJ. Br. Med. J. 311, 42–45. doi: 10.1136/bmj.311.6996.42, PMID: 7613329 PMC2550091

[ref44] Pope C. Ziebland S. Mays N. (2000). Qualitative research in health care–analysing qualitative data (reprinted from qualitative research in health care). Br. Med. J. 320, 114–116. doi: 10.1136/bmj.320.7227.114, PMID: 10625273 PMC1117368

[ref45] Powell L. Edwards K. M. Mcgreevy P. Bauman A. Podberscek A. Neilly B.. (2019). Companion dog acquisition and mental well-being: a community-based three-arm controlled study. BMC Public Health 19:1428. doi: 10.1186/s12889-019-7770-5, PMID: 31684914 PMC6829935

[ref46] Ritchie H. Roser M. (2018). Mental Health. *Our World in Data*.

[ref47] Rodriguez K. E. Herzog H. Gee N. R. (2021). Variability in human-animal interaction research. Front. Vet. Sci. 7:619600. doi: 10.3389/fvets.2020.619600 33521092 PMC7843787

[ref48] Ron D. H. Jakob B. B. Dennis A. R. Karen L. S. David C. (2009). Development of physical and mental health summary scores from the patient-reported outcomes measurement information system (PROMIS) global items. Qual. Life Res. 18:873. doi: 10.1007/s11136-009-9496-9 19543809 PMC2724630

[ref49] Ryan R. M. Deci E. L. (2001). On happiness and human potentials: a review of research on hedonic and Eudaimonic well-being. Annu. Rev. Psychol. 52, 141–166. doi: 10.1146/annurev.psych.52.1.141, PMID: 11148302

[ref50] Ryff C. D. (1989). Happiness is everything, or is it? Explorations on the meaning of psychological well-being. J. Pers. Soc. Psychol. 57, 1069–1081. doi: 10.1037/0022-3514.57.6.1069

[ref51] Saunders J. Parast L. Babey S. H. Miles J. V. (2017). Exploring the differences between pet and non-pet owners: implications for human–animal interaction research and policy. PLoS One 12, 1–15. doi: 10.1371/journal.pone.0179494 PMC5482437 28644848

[ref52] Saunders B. Sim J. Kingstone T. Baker S. Waterfield J. Bartlam B.. (2018). Saturation in qualitative research: exploring its conceptualization and operationalization. Qual. Quant. 52, 1893–1907. doi: 10.1007/s11135-017-0574-8, PMID: 29937585 PMC5993836

[ref53] Schubert T. Eloo R. Scharfen J. Morina N. (2020). How imagining personal future scenarios influences affect: systematic review and meta-analysis. Clin. Psychol. Rev. 75:101811. doi: 10.1016/j.cpr.2019.101811, PMID: 31884148

[ref54] Sharpley C. Veronese N. Smith L. López-Sánchez G. F. Bitsika V. Demurtas J.. (2020). Pet ownership and symptoms of depression: a prospective study of older adults. J. Affect. Disord. 264, 35–39. doi: 10.1016/j.jad.2019.11.134, PMID: 31846900

[ref55] Spitznagel M. B. Anderson J. R. Marchitelli B. Sislak M. D. Bibbo J. Carlson M. D. (2021). Owner quality of life, caregiver burden and anticipatory grief: how they differ, why it matters. Vet. Rec. 188:e74. doi: 10.1002/vetr.74 33960467

[ref56] Staats S. Wallace H. Anderson T. (2008). Reasons for Companion Animal Guardianship (Pet Ownership) from Two Populations. Great Britain: White Horse Press.

[ref57] Stern S. L. Donahue D. A. Allison S. Hatch J. P. Lancaster C. L. Benson T. A.. (2013). Potential benefits of canine companionship for military veterans with posttraumatic stress disorder (PTSD). Soc. Anim. 21, 568–581. doi: 10.1163/15685306-12341286

[ref58] Sundman A.-S. Van Poucke E. Svensson Holm A.-C. Olsen Faresjö Å. Theodorsson E. Jensen P.. (2019). Long-term stress levels are synchronized in dogs and their owners. Sci. Rep. 9:7391. doi: 10.1038/s41598-019-43851-x, PMID: 31171798 PMC6554395

[ref59] Tower R. B. Nokota M. (2006). Pet companionship and depression: results from a United States internet sample. Anthrozoös 19, 50–64. doi: 10.2752/089279306785593874

[ref60] Westgarth C. Christley R. M. Christian H. E. (2014). How might we increase physical activity through dog walking?: a comprehensive review of dog walking correlates. Int. J. Behav. Nutr. Phys. Act. 11:83. doi: 10.1186/1479-5868-11-83 25142228 PMC4261546

[ref62] Westgarth C. Christley R. M. Marvin G. Perkins E. (2017). I walk my dog Because it makes me happy: A qualitative study to understand why dogs motivate walking and improved health. Int. J. of Environ. Res. Public Health 14:936. doi: 10.3390/ijerph14080936 PMC5580638 28825614

[ref63] Westgarth C. Christley R. M. Marvin G. Perkins E. (2019). The responsible dog owner: The construction of responsibility. Anthrozoös 32, 631–646. doi: 10.1080/08927936.2019.1645506

[ref64] Westgarth C. Christley R. M. Marvin G. Perkins E. (2021). Functional and recreational dog walking practices in the UK. Health Promot. Int. 36, 109–119. doi: 10.1093/heapro/daaa051, PMID: 32361764 PMC7954209

[ref65] Wheeler E. A. Faulkner M. E. (2015). The "pet effect": physiological calming in the presence of canines. Soc. Anim. 23, 425–438. doi: 10.1163/15685306-12341374

[ref66] While A. (2017). Pet dogs as promoters of wellbeing. Br. J. Community Nurs. 22, 332–336. doi: 10.12968/bjcn.2017.22.7.332, PMID: 28686101

[ref67] WHO (2018a). Global Health Estimates 2016: Disease Burden by Cause, Age, Sex, by Country and by Region, 2000–2016. WHO: Geneva.

[ref68] WHO (2018b). Mental Health: Strengthening Our Response [online]. Geneva: World Health Organisation.

[ref69] Wong P. W. C. Yu R. W. M. Ngai J. T. K. (2019). Companion animal ownership and human well-being in a Metropolis-The case of Hong Kong. Int. J. Env. Res. Public Health 16:1729. doi: 10.3390/ijerph16101729 PMC6571622 31100852

[ref70] Wood L. J. Giles-Corti B. Bulsara M. K. Bosch D. A. (2007). More than a furry companion: The ripple effect of companion animals on neighborhood interactions and sense of community. Soc. Anim. 15, 43–56. doi: 10.1163/156853007x169333

[ref71] Young J. Bowen-Salter H. O’dwyer L. Stevens K. Nottle C. Baker A. (2020). A qualitative analysis of pets as suicide protection for older people. Anthrozoös 33, 191–205. doi: 10.1080/08927936.2020.1719759

[ref72] Zimolag U. Krupa T. (2009). Pet ownership as a meaningful community occupation for people With serious mental illness. Am. J. Occup. Ther. 63, 126–137. doi: 10.5014/ajot.63.2.126, PMID: 19432050

