# Supplementary material for: Dogs and the Good Life: A Cross-Sectional Study of the Association Between the Dog–Owner Relationship and Owner Mental Wellbeing
Source: Front Psychol. 2022 Jul 18;13:903647. doi: 10.3389/fpsyg.2022.903647 (PMC9341998; doi:10.3389/fpsyg.2022.903647)
Supplement: Supplementary file 1 [file Data_Sheet_1.docx]

Supplementary Material

Dogs and the Good Life: A cross sectional study of the association between the dog-owner relationship and owner mental wellbeing

# Supplementary Tables

## Quantitative methodology

The tables below explain the scores given in the relationship and mental health scales (MDORS and PROMIS scales).

**Supplementary Table 1:** Dog-owner relationship categories and maximum scores explained (MDORS scale).

| **MDORS category** | **Number of questions** | **Maximum Score** | **Interpretation of maximum score** |
| --- | --- | --- | --- |
| Pet-owner interaction | 9 | 45 | Maximum pet-owner interaction |
| Perceived emotional closeness | 10 | 50 | Maximum emotional closeness between the pet and the owner as perceived by the owner |
| Perceived costs | 9 | 45 | Minimum burden as considered by the owner |
| Total Relationship | 28 | 140 | Closest relationship possible between the pet and the owner |

**Supplementary Table 2:** Mental health outcomes categories and maximum scores explained (PROMIS scale).

| **PROMIS category** | **Number of questions** | **Maximum Score** | **Interpretation of maximum score** |
| --- | --- | --- | --- |
| Anxiety | 8 | 40 | Higher anxiety levels – poorer mental health in terms of anxiety​ |
| Depression | 8 | 40 | Higher depression levels – poorer mental health in terms of depression​ |
| Emotional support | 4 | 20 | Minimum emotional support – poorer mental health in terms of emotional support​ |
| Companionship | 4 | 20 | Minimum companionship – poorer mental health in terms of companionship​ |

## Cumulative tables of participants’ mental health responses

The tables below show the calculations of PROMIS mental health scores. The numbers in brackets indicate the score given for each answer.

**Supplementary Table 3:** Cumulative table of the responses given by the participants concerning the questions of the PROMIS item bank v1.0 Anxiety Scale Form 8a.

| In the past 7 days… | Never (1) | Rarely (2) | Sometimes (3) | Often (4) | Always (5) | Missing |
| --- | --- | --- | --- | --- | --- | --- |
| I felt fearful | 961 | 355 | 268 | 75 | 24 | 10 |
| I found it hard to concentrate on anything other than my anxiety | 1007 | 288 | 251 | 114 | 27 | 6 |
| My worries overwhelmed me | 915 | 351 | 262 | 123 | 37 | 5 |
| I felt uneasy | 652 | 473 | 369 | 156 | 36 | 7 |
| I felt nervous | 674 | 478 | 355 | 148 | 31 | 7 |
| I felt like I needed help for my anxiety | 1064 | 249 | 213 | 120 | 42 | 5 |
| I felt anxious | 693 | 395 | 375 | 178 | 48 | 4 |
| I felt tense | 497 | 461 | 466 | 216 | 49 | 4 |

**Supplementary Table 4:** Cumulative table of the responses given by the participants concerning the questions of the PROMIS item bank v1.0 Depression Scale Form 8b.

| In the past 7 days… | Never (1) | Rarely (2) | Sometimes (3) | Often (4) | Always (5) | Missing |
| --- | --- | --- | --- | --- | --- | --- |
| I felt worthless | 938 | 320 | 280 | 111 | 36 | 8 |
| I felt helpless | 914 | 343 | 289 | 110 | 34 | 3 |
| I felt depressed | 730 | 397 | 347 | 147 | 71 | 1 |
| I felt hopeless | 930 | 315 | 281 | 113 | 42 | 12 |
| I felt like a failure | 875 | 326 | 291 | 141 | 55 | 5 |
| I felt unhappy | 372 | 535 | 539 | 183 | 62 | 2 |
| I felt that I had nothing to look forward to | 952 | 337 | 239 | 116 | 45 | 4 |
| I felt that nothing could cheer me up | 1042 | 311 | 228 | 85 | 27 | 0 |

**Supplementary Table 5:** Cumulative table of the responses given by the participants concerning the questions of the PROMIS item bank v2.0 Emotional Support Scale Form 4a.

|  | Always (1) | Usually (2) | Sometimes (3) | Rarely (4) | Never (5) | Missing |
| --- | --- | --- | --- | --- | --- | --- |
| I have someone who will listen to me when I need to talk | 654 | 552 | 301 | 150 | 34 | 2 |
| I have someone to confide in or talk about myself or my problems | 657 | 537 | 298 | 147 | 49 | 5 |
| I have someone who makes me feel appreciated | 671 | 525 | 320 | 135 | 40 | 2 |
| I have someone to talk when I have a bad day | 735 | 509 | 275 | 130 | 43 | 1 |

**Supplementary Table 6:** Cumulative table of the responses given by the participants concerning the questions of the PROMIS item bank v2.0 Companionship Scale Form 4a.

|  | Always (1) | Usually (2) | Sometimes (3) | Rarely (4) | Never (5) | Missing |
| --- | --- | --- | --- | --- | --- | --- |
| Do you somebody with whom to have fun? | 623 | 602 | 350 | 94 | 22 | 2 |
| Do you somebody with whom to relax? | 687 | 544 | 289 | 126 | 44 | 3 |
| Do you somebody with whom you can do something enjoyable? | 659 | 599 | 318 | 92 | 20 | 5 |
| Can you find companionship when you want it? | 628 | 617 | 313 | 112 | 22 | 1 |

## Cumulative tables of participants’ dog-owner relationship scale responses

The tables below show the calculations of MDORS scores. The numbers in brackets indicate the score given for each answer.

**Supplementary Table 7:** Cumulative table of the responses given by the participants concerning the questions of the MDORS Pet-owner interaction scale.

|  | Never (1) | Once a month (2) | Once a week (3) | Once every few days (4) | At least once a day (5) | Missing |
| --- | --- | --- | --- | --- | --- | --- |
| How often do you kiss your dog? | 132 | 12 | 36 | 128 | 1384 | 1 |
| How often do you play games with your dog? | 26 | 12 | 53 | 226 | 1374 | 2 |
| How often do you have your dog with you while relaxing i.e. watching TV? | 8 | 2 | 3 | 21 | 1658 | 1 |
| How often do you give your dog food treats? | 28 | 42 | 96 | 366 | 1158 | 3 |
| How often do you take your dog in the car? | 60 | 268 | 293 | 608 | 458 | 6 |
| How often do you hug your dog? | 37 | 10 | 18 | 81 | 1540 | 7 |
| How often do you groom your dog? | 85 | 489 | 457 | 481 | 177 | 4 |
|  | Never (1) | A couple of times a year (2) | Once a month (3) | Once a fortnight (4) | Once a week (5) | Missing |
| How often do you buy you dog presents? | 66 | 612 | 529 | 236 | 249 | 1 |
| How often do you take your dog to visit people? | 115 | 227 | 278 | 272 | 795 | 6 |

**Supplementary Table 8:** Cumulative table of the responses given by the participants concerning the questions of the MDORS Perceived Emotional Closeness scale.

|  | Strongly disagree (1) | Disagree (2) | Neither agree nor disagree (3) | Agree (4) | Strongly agree (5) | Missing |
| --- | --- | --- | --- | --- | --- | --- |
| My dog gives me a reason to get up in the morning | 4 | 29 | 169 | 545 | 944 | 2 |
| I wish my dog and I never had to be apart | 12 | 102 | 273 | 520 | 786 | 0 |
| I would like to have my dog near me all the time | 8 | 84 | 234 | 531 | 832 | 4 |
| If everyone else left me, my dog would still be there for me | 4 | 14 | 76 | 351 | 1247 | 1 |
| My dog helps me get through tough times | 3 | 15 | 123 | 458 | 1090 | 4 |
| My dog provides me with constant companionship | 2 | 12 | 42 | 371 | 1265 | 1 |
| My dog is there whenever I need to be comforted | 1 | 18 | 98 | 373 | 1203 | 0 |
| My dog is constantly attentive to me | 8 | 58 | 208 | 632 | 785 | 2 |
|  | Never (1) | Once a year (2) | Once a month (3) | Once a week (4) | Once a day (5) | Missing |
| How often do you tell you dog things you do not tell to anyone? | 503 | 66 | 213 | 460 | 444 | 7 |
|  | Very untraumatic (1) | Untraumatic (2) | Neither traumatic nor untraumatic (3) | Traumatic (4) | Very traumatic (5) | Missing |
| How traumatic do you think it will be for you when your dog dies? | 9 | 3 | 29 | 277 | 1373 | 2 |

**Supplementary Table 9:** Cumulative table of the responses given by the participants concerning the questions of the MDORS Perceived Costs scale.

|  | Strongly agree (1) | Agree (2) | Neither agree nor disagree (3) | Disagree (4) | Strongly disagree (5) | Missing |
| --- | --- | --- | --- | --- | --- | --- |
| There are major aspects of owning a dog that I do not like | 32 | 126 | 197 | 600 | 735 | 3 |
| My dog makes too much mess | 10 | 85 | 206 | 680 | 711 | 1 |
| It bothers me that my dog stops me doing things I enjoyed before I owned it | 8 | 51 | 168 | 586 | 880 | 0 |
| It is annoying that sometimes I have to change my plans because of my dog | 9 | 153 | 276 | 620 | 635 | 0 |
| My dog costs too much money | 7 | 64 | 199 | 617 | 805 | 1 |
|  | Once a day (1) | Once a week (2) | Once a month (3) | Once a year (4) | Never (5) | Missing |
| How often do you feel that looking after your dog is a chore? | 20 | 101 | 199 | 184 | 1180 | 9 |
| How often do your dog stop you doing things you want to? | 19 | 94 | 324 | 355 | 899 | 2 |
| How often do you feel that having a dog is more trouble than it is worth? | 8 | 23 | 61 | 127 | 1473 | 1 |
|  | Very hard (1) | Hard (2) | Neither hard nor easy (3) | Easy (4) | Very Easy (5) | Missing |
| How hard is to look after your dog? | 11 | 140 | 638 | 596 | 307 | 1 |

# Questionnaire

**DOG RELATED QUESTIONS**

1. How many dogs do you own?

- 1
- 2
- 3
- More than 3

1. Why did you choose to get a dog? (Please indicate all that apply)

- Companionship
- Protection
- Interest/hobby
- Gift
- To show
- To breed
- Exercise
- Working dog
- Always had a dog
- Family member wanted a dog
- Other – Please describe

__________________________________________________________________

**Please complete the following questions for the dog you feel emotionally closest to.**

1. How old is your dog?

_____________________________________________________________________

1. What sex is your dog?

- Male
- Female

1. Is your dog neutered?

- Yes
- No

1. What is your dog’s breed?

- Pure Breed
- Known Cross – Please describe
- Unknown

1. How long have you owned your dog?

____________________________________________________________________

1. Where did you get the dog?

- From the person who bred it
- Rescue centre
- Through a friend
- From a family member
- Found as stray
- Gift
- Pet shop
- Bred it ourselves
- Other – Please indicate _________________________________________________

1. Where does your dog spend most of the day?

- Yard
- Garden
- Some particular rooms at the house
- All rooms of the house

1. What size is your dog?

- Toy (eg Chihuahua)
- Small (eg Terrier, Pug)
- Medium (eg Spaniel, Collie)
- Large (eg Labrador, German Shepherd)
- Giant (eg St Bernard, Great Dane)

1. Is your dog overweight?

- Yes
- No

1. What does your dog’s diet consist of? (Please indicate all that apply)

- Pre-prepared moist pet foods (eg Canned/pouch foods)
- Home prepared foods (eg fresh cooked meat and/or vegetables)
- Raw meat
- Pre-prepared complete dry foods
- Mixer biscuits
- Table scraps
- Purchased snacks and treats
- Other – Please indicate _______________________________________________________________

1. How much do you spend on dog food for this dog per month?

_____________________________________________________________________

1. Are you the primary caretaker for this dog? (Do the majority of walking, feeding, grooming etc).

- Yes
- No

**DOG-OWNER RELATIONSHIP QUESTIONS**

1. How hard is it to look after your dog?

- Very hard
- Hard
- Neither hard nor easy
- Easy
- Very easy

1. My dog gives me a reason to get up in the morning

- Strongly agree
- Agree
- Neither agree nor disagree
- Disagree
- Strongly disagree

1. There are major aspects of owning a dog I don’t like.

- Strongly agree
- Agree
- Neither agree nor disagree
- Disagree
- Strongly disagree

1. How often do you kiss your dog?

- Never
- Once a month
- Once a week
- Once every few days
- At least once a day

1. I wish my dog and I never had to be apart

- Strongly agree
- Agree
- Neither agree nor disagree
- Disagree
- Strongly disagree

1. My dog makes too much mess.

- Strongly agree
- Agree
- Neither agree nor disagree
- Disagree
- Strongly disagree

1. How often do you play games with your dog?

- At least once a day
- Once every few days
- Once a week
- Once a month
- Never

1. How often do you give your dog treats?

- Never
- Once a month
- Once a week
- Once every few days
- At least once a day

1. It bothers me that my dog stops me doing things I enjoyed before I owned it

- Strongly agree
- Agree
- Neither agree nor disagree
- Disagree
- Strongly disagree

1. How often do you spend time enjoying watching your dog?

- At least once a day
- Once every few days
- Once a week
- Once a month
- Never

1. It is annoying that sometimes I have to change my plans because of my dog

- At least once a day
- Once every few days
- Once a week
- Once a month
- Never

1. My dog costs me too much money

- Strongly agree
- Agree
- Neither agree nor disagree
- Disagree
- Strongly disagree

1. How often do you buy your dog presents?

- Once a week
- Once a fortnight
- Once a month
- A couple of times a year
- Never

1. How often do you tell your dog things you do not tell anyone else?

- Once a day
- Once a week
- Once a month
- Once a year
- Never

1. How often do you feel that looking after your dog is a chore?

- Once a day
- Once a week
- Once a month
- Once a year
- Never

1. How often do you talk to your dog?

- At least once a day
- Once every few days
- Once a week
- Once a month
- Never

1. How often does your dog stop you doing things you want to?

- Once a day
- Once a week
- Once a month
- Once a year
- Never

1. I would like to have my dog near me all the time

- Strongly agree
- Agree
- Neither agree nor disagree
- Disagree
- Strongly disagree

1. If everyone else left me, my dog would still be there for me

- Strongly agree
- Agree
- Neither agree nor disagree
- Disagree
- Strongly disagree

1. How often do you feel that having a dog is more trouble than it is worth?

- Once a day
- Once a week
- Once a month
- Once a year
- Never

1. My dog helps me get through tough times

- Strongly agree
- Agree
- Neither agree nor disagree
- Disagree
- Strongly disagree

1. How often do you cuddle your dog?

- At least once a day
- Once every few days
- Once a week
- Once a month
- Never

1. My dog provides me with constant companionship

- Strongly agree
- Agree
- Neither agree nor disagree
- Disagree
- Strongly disagree

1. How often do you have your dog with you while relaxing i.e. watching TV?

- At least once a day
- Once every few days
- Once a week
- Once a month
- Never

1. My dog is here whenever I need to be comforted

- Strongly agree
- Agree
- Neither agree nor disagree
- Disagree
- Strongly disagree

1. How traumatic do you think it will be for you when your dog dies?

- Very traumatic
- Traumatic
- Neither traumatic nor untraumatic
- Untraumatic
- Very untraumatic

1. How often do you pet your dog?

- At least once a day
- Once every few days
- Once a week
- Once a month
- Never

1. How often do you take your dog to visit people?

- Once a week
- Once a fortnight
- Once a month
- A couple of times a year
- Never

1. How often do you give your dog food treats?

- At least once a day
- Once every few days
- Once a week
- Once a month
- Never

1. How often do you take your dog in the car?

- At least once a day
- Once every few days
- Once a week
- Once a month
- Never

1. How often do you hug your dog?

- At least once a day
- Once every few days
- Once a week
- Once a month
- Never

1. How often do you groom your dog?

- At least once a day
- Once every few days
- Once a week
- Once a month
- Never

1. My dog is constantly attentive to me

- Strongly agree
- Agree
- Neither agree nor disagree
- Disagree
- Strongly disagree

**OWNER HEALTH RELATED QUESTIONS**

1. In general. Would you say your health is:

- Excellent
- Very good
- Good
- Fair
- Poor

1. In general, would you say your quality of life is:

- Excellent
- Very good
- Good
- Fair
- Poor

1. In general, how would you rare your physical health?

- Excellent
- Very good
- Good
- Fair
- Poor

1. In general, how would you rate your mental health, including your mood and your ability to think?

- Excellent
- Very good
- Good
- Fair
- Poor

1. In general, how would you rate your satisfaction with your social activities and relationships?

- Excellent
- Very good
- Good
- Fair
- Poor

1. In general, please rate how well you carry out your usual social activities and roles. (This includes activities at home, at work and in your community, and responsibilities as a parent, child, spouse, employee, friend, etc.)

- Excellent
- Very good
- Good
- Fair
- Poor

1. To what extend are you able to carry out your everyday physical activities such as walking, climbing stairs, carrying groceries, or moving a chair?

- Completely
- Mostly
- Moderately
- A little
- Not at all

1. In the past 7 days, how often have you been bothered by emotional problems such as feeling anxious, depressed or irritable?

- Never
- Rarely
- Sometimes
- Often
- Always

1. How would you rate your pain on average?

- 0 (No pain)
- 1
- 2
- 3
- 4
- 5
- 6
- 7
- 8
- 9
- 10 (Worst pain imaginable)

1. In the past 7 days, I felt worthless

- Never
- Rarely
- Sometimes
- Often
- Always

1. In the past 7 days, I felt helpless

- Never
- Rarely
- Sometimes
- Often
- Always

1. In the past 7 days, I felt depressed

- Never
- Rarely
- Sometimes
- Often
- Always

1. In the past 7 days, I felt hopeless

- Never
- Rarely
- Sometimes
- Often
- Always

1. In the past 7 days, I felt like a failure

- Never
- Rarely
- Sometimes
- Often
- Always

1. In the past 7 days, I felt unhappy

- Never
- Rarely
- Sometimes
- Often
- Always

1. In the past 7 days, I felt that I had nothing to look forward to

- Never
- Rarely
- Sometimes
- Often
- Always

1. In the past 7 days, I felt that nothing could cheer me up

- Never
- Rarely
- Sometimes
- Often
- Always

1. In the past 7 days, I felt fearful

- Never
- Rarely
- Sometimes
- Often
- Always

1. In the past 7 days, I found it hard to focus on anything other than my anxiety

- Never
- Rarely
- Sometimes
- Often
- Always

1. In the past 7 day, my worries overwhelmed me

- Never
- Rarely
- Sometimes
- Often
- Always

1. In the past 7 days, I felt uneasy

- Never
- Rarely
- Sometimes
- Often
- Always

1. In the past 7 days, I felt nervous

- Never
- Rarely
- Sometimes
- Often
- Always

1. In the past 7 days, I felt like I needed help for my anxiety

- Never
- Rarely
- Sometimes
- Often
- Always

1. In the past 7 days, I felt anxious

- Never
- Rarely
- Sometimes
- Often
- Always

1. In the past 7 days, I felt tense

- Never
- Rarely
- Sometimes
- Often
- Always

1. I will have someone who will listen to me when I need to talk

- Never
- Rarely
- Sometimes
- Often
- Always

1. I have someone to confide in or talk to about myself or my problems

- Never
- Rarely
- Sometimes
- Often
- Always

1. I have someone who makes me feel appreciated

- Never
- Rarely
- Sometimes
- Often
- Always

1. I have someone to talk with when I have a bad day

- Never
- Rarely
- Sometimes
- Often
- Always

1. Do you have someone with whom to have fun?

- Never
- Rarely
- Sometimes
- Often
- Always

1. Do you have someone with whom to relax?

- Never
- Rarely
- Sometimes
- Often
- Always

1. Do you have someone with whom you can do something enjoyable?

- Never
- Rarely
- Sometimes
- Often
- Always

1. Can you find companionship when you want it?

- Never
- Rarely
- Sometimes
- Often
- Always

1. Describe any ways that you think owning a dog improves your personal mental health:

__________________________________________________________________________

1. Describe any ways that you think owning a dog makes your personal mental health worse:

__________________________________________________________________________

1. What are the biggest challenges you face in caring for your dog?

__________________________________________________________________________

1. A) Have you ever been diagnosed with a mental health problem?

- Yes
- No

B) If yes, with what mental health problem were you diagnosed?

- Anxiety and panic attacks
- Bipolar disorder
- Depression
- Eating disorder
- Obsessive – compulsory disorder (OCD)
- Post-Traumatic Stress Disorder (PTSD)
- Psychosis
- Schizophrenia
- Self-harm
- Suicidal feelings
- Other – Please indicate

_______________________________________________________________

C) If yes, did you take / are you currently taking any medication concerning your mental health problem?

- Yes
- No

1. Do you have any physical disability or chronic disease?

- Yes
- No

1. Who, if anyone, usually walks or jogs with your dog? (Please indicate all that apply)

- No one
- Me
- Spouse/Partner
- Child
- Shared across household members
- Professional dog walker
- Friend or other family member
- Other – Please specify

1. In a USUAL WEEK, how many times, if any, and for how many minutes, do you walk, jog/run or cycle WITH YOUR DOG for recreation health or fitness or as a means of transport, (such as going to or from work, walking to the shop, or walking to public transport)?

Times per week (if none, put 0):________________________________________________

Minutes per week (e.g. 2 times for 40 minutes = 80 minutes):_________________________

1. In a USUAL WEEK, how many times, if any, and for how many minutes, do you personally Walk WITHOUT YOUR DOG as a means of transport, (such as going to or from work, walking to the shop, or walking to public transport)?

Times per week (if none, put 0):________________________________________________

Minutes per week (e.g. 2 times for 40 minutes = 80 minutes):_________________________

1. In a USUAL WEEK, how many times, if any, and for how many minutes, do you personally Walk WITHOUT YOUR DOG for recreation, health or fitness?

Times per week (if none, put 0):________________________________________________

Minutes per week (e.g. 2 times for 40 minutes = 80 minutes):_________________________

1. In a USUAL WEEK, how many times, if any, and for how many minutes, do you personally do other moderate intensity physical activities WITHOUT YOUR DOG such as swimming, cycling, social tennis, golf or heavy gardening. Moderate intensity physical activities do not make you breathe harder or puff and pant. Do not include walking.

Times per week (if none, put 0):________________________________________________

Minutes per week (e.g. 2 times for 40 minutes = 80 minutes):_________________________

1. In a USUAL WEEK, how many times, if any, and for how many minutes, do you personally do any vigorous intensity activities WITHOUT YOUR DOG such as jogging, aerobics, mountain biking, competitive cycling or tennis. Vigorous intensity physical activities make you breathe harder or puff and pant.
   Do not include walking or moderate activities.

Times per week (if none, put 0):________________________________________________

Minutes per week (e.g. 2 times for 40 minutes = 80 minutes):_________________________

**QUESTIONS ABOUT YOURSELF**

1. How old are you?

- 18-24 years old
- 25-34 years old
- 35-44 years old
- 45-54 years old
- 55-64 years old
- >65 years old

1. Are you:

- Male
- Female
- Other

1. What is your weight? (in kg or stones)

__________________________________________________________________________

1. What is your height?

__________________________________________________________________________

1. How many people live in the household?

- 1
- 2
- 3
- 4+

1. Are there any children under the age of 16 living in the household?

- Yes
- No

1. What is your marital status?

- Married
- Living with partner
- Divorced/Separated
- Widowed
- Single, never married

1. What is the highest educational or school qualification that you have obtained?

- University Higher Degree (e.g. MSc, PhD)
- First degree level qualification including foundation degrees. Graduate membership of a professional institute, PGCE
- Diploma in Higher Education
- Teaching qualification (excluding PGCE)
- Nursing or other medical qualification not yet mentioned
- A Level
- Welsh Baccalaureate
- International Baccalaureate
- AS Level
- Higher Grade/Advanced Higher (Scotland)
- Certificate of sixth year studies
- GCSE/O Level
- GSE
- Standard/Ordinary (O) Grade / Lower (Scotland)
- Other school (including School leaving exam certificate or matriculation)
- None of the above

1. Do you have a full-time or part-time job (including full-time student) of any kind?

- Yes, work for wage, payment or profit
- Yes, unpaid work
- Retired
- Home duties
- No, do not have a job

1. Which country do you live in?

__________________________________________________________________________
